# Extracellular vesicles from human airway basal cells respond to cigarette smoke extract and affect vascular endothelial cells

**DOI:** 10.1038/s41598-021-85534-6

**Published:** 2021-03-17

**Authors:** Ashish Saxena, Matthew S. Walters, Jae-Hung Shieh, Ling-Bo Shen, Kazunori Gomi, Robert J. Downey, Ronald G. Crystal, Malcolm A. S. Moore

**Affiliations:** 1 Department of Cell Biology, Sloan Kettering Institute, Memorial Sloan Kettering Cancer Center, New York, NY USA; 2 Department of Genetic Medicine, Weill Cornell Medicine, New York, NY USA; 3 Thoracic Service, Department of Surgery, Memorial Sloan Kettering Cancer Center, New York, NY USA; 4 Present Address: Department of Medicine, Division of Hematology and Medical Oncology, Weill Cornell Medicine, New York, NY USA; 5 Present Address: Department of Medicine, Division of Pulmonary, Critical Care and Sleep Medicine, University of Oklahoma Health Sciences Center, Oklahoma City, OK USA; 6 Present Address: Department of Pathology and Laboratory Medicine, Weill Cornell Medicine, New York, NY USA; 7 Present Address: Cell Therapy and Cell Engineering Facility, Memorial Sloan Kettering Cancer Center, New York, NY USA; 8 Present Address: Department of Physiology and Biophysics, Weill Cornell Medicine, New York, NY USA

**Keywords:** Respiratory tract diseases, Cell biology, Cell signalling

## Abstract

The human airway epithelium lining the bronchial tree contains basal cells that proliferate, differentiate, and communicate with other components of their microenvironment. One method that cells use for intercellular communication involves the secretion of exosomes and other extracellular vesicles (EVs). We isolated exosome-enriched EVs that were produced from an immortalized human airway basal cell line (BCi-NS1.1) and found that their secretion is increased by exposure to cigarette smoke extract, suggesting that this stress stimulates release of EVs which could affect signaling to other cells. We have previously shown that primary human airway basal cells secrete vascular endothelial growth factor A (VEGFA) which can activate MAPK signaling cascades in endothelial cells via VEGF receptor–2 (VEGFR2). Here, we show that exposure of endothelial cells to exosome-enriched airway basal cell EVs promotes the survival of these cells and that this effect also involves VEGFR2 activation and is, at least in part, mediated by VEGFA present in the EVs. These observations demonstrate that EVs are involved in the intercellular signaling between airway basal cells and the endothelium which we previously reported. The downstream signaling pathways involved may be distinct and specific to the EVs, however, as increased phosphorylation of Akt, STAT3, p44/42 MAPK, and p38 MAPK was not seen following exposure of endothelial cells to airway basal cell EVs.

## Introduction

The human airway epithelium lining the bronchial tree comprises a number of different cell types, including ciliated and secretory cells, intermediate or undifferentiated cells, and basal cells. Airway epithelium basal cells reside near the basement membrane and function as stem or progenitor cells, capable of proliferating and differentiating into the other cell types during normal epithelial regeneration and repair^1,2^. Stress, such as cigarette smoke or inflammatory stimuli, can alter the normal differentiation process, resulting in a disordered airway epithelium. The biology of basal cells is therefore of interest to better understand both normal airway epithelium functioning and a number of disease states of the airways, such as chronic obstructive pulmonary disease (COPD) and bronchogenic carcinoma.

One important aspect of basal cell biology involves communication with other cells within the microenvironment. Exosomes are a subclass of secreted extracellular vesicles (EVs) measuring 40–150 nm in diameter that are released by cells, through fusion of cytoplasmic multivesicular bodies with the cell membrane, into the pericellular milieu and into circulating networks such as the peripheral bloodstream. Exosomes contain a variety of proteins, lipids, mRNAs, and microRNAs that are representative of the molecules within the cell that produced them. The physiologic functions of exosomes depend on their cell of origin as well as their composition. In general, they are believed to function in intercellular signaling by trafficking macromolecules from one cell to another and to distant microenvironments^3,4^.

Here we report the isolation of exosome-enriched EVs secreted from an immortalized human airway basal cell line (BCi-NS1.1). This cell line was generated from primary human airway basal cells and was immortalized via infection with a retrovirus expressing human telomerase (hTERT). The resulting cell line retains characteristics of the original primary cells for > 40 passages and can differentiate into secretory, goblet, Clara, and ciliated cells^5^. We examined the effect of an external stimuli, cigarette smoke extract (CSE), on the secretion of EVs from this cell line, as well as the effect that these EVs had on other cell types similar to those that could be found in the tissue microenvironment, such as endothelial cells. Although there is a lack of physical contact between airway epithelial basal cells and endothelial cells within the local airway microenvironment, prior studies have demonstrated that the airway epithelium is capable of signaling to non-epithelial cell types in the tissue microenvironment in a paracrine manner^6^. We hypothesized that EVs secreted from airway epithelial basal cells may also function in this paracrine manner and focused on examining signaling to endothelial cells specifically, based on prior studies from our group^5,7–9^. Finally, we characterized candidate downstream molecular mechanisms that may be involved in the effects of airway basal cell EVs on endothelial cells.

## Materials and methods

### Cell culture

The origin and development of the BCi-NS1.1 cell line has been described previously^5^. Briefly, this cell line was generated from primary human airway basal cells obtained by brushing the airway epithelium of healthy nonsmokers and was immortalized via infection with a retrovirus expressing human telomerase (hTERT). The resulting cell line, which retains characteristics of the original primary cells for > 40 passages, has a multipotent differentiation capacity into secretory, goblet, Clara, and ciliated cells.

BCi-NS1.1 cells were incubated at 37 °C and maintained in bronchial epithelial cell growth medium (BEGM; Lonza, Basel, Switzerland), which comprises bronchial epithelial cell basal medium (BEBM) supplemented with the following growth factors: bovine pituitary extract (BPE), hydrocortisone, human epidermal growth factor, epinephrine, transferrin, insulin, retinoic acid, triiodothyronine, and gentamicin/amphotericin-B. Once the BCi-NS1.1 cells reached 70%-80% confluence, the fully supplemented BEGM was removed and the cells were washed in phosphate-buffered saline (PBS). They were then incubated at 37 °C for 48 h in BEGM lacking BPE, after which time EVs were isolated from the resultant conditioned media. After removal of conditioned media, adherent cells were washed in PBS and then treated with Trypsin–EDTA (Corning Life Sciences, Corning, NY, USA) to detach them. The cells were then resuspended in HEPES buffer with fetal bovine serum (FBS) (Gemini Bio, West Sacramento, CA, USA). The cells were pelleted by centrifugation at 250X g for 5 min at 4 °C, washed once with PBS, and then resuspended in BEGM. A 10-uL aliquot of these resuspended cells was then mixed with Trypan Blue (Corning Life Sciences), and the number of live cells was counted on a hemocytometer. The remaining cells were again pelleted as above, washed in PBS, and then pelleted again. The resulting cell pellets were then frozen in liquid nitrogen and stored at -70 °C. Once these cell pellets were thawed, they were dissolved and lysed in radioimmunoprecipitation (RIPA) lysis buffer with protease and phosphatase inhibitor cocktail (Sigma-Aldrich, St. Louis, MO, USA) for 30 min at 4 °C. Cell debris was pelleted, and the supernatant was removed and used for immunoblotting.

H1975 non-small cell lung cancer cells were obtained from the American Type Culture Collection (Manassas, VA, USA) and were grown in M5 medium consisting of DMEM-F12 supplemented with 25 mM HEPES and 2.2 g/L sodium bicarbonate (Media Preparation Core Facility, Memorial Sloan Kettering Cancer Center). N417 small cell lung cancer cells were a gift from Dr. Govindaswami Ragupathi (Memorial Sloan Kettering Cancer Center) and were grown in RPMI 1640 media (Corning Life Sciences). Both M5 and RPMI media were supplemented with 10% (v/v) FBS (Gemini Bio), 2 mM L-glutamine, and 500 U/mL penicillin and streptomycin. Once cells became 70–80% confluent, this media was removed and the cells were washed in PBS and then incubated for 48 h in the appropriate media without FBS but with 1X insulin-transferrin-selenium mix (Thermo Fisher Scientific, Waltham, MA, USA). This conditioned media was then collected for isolation of EVs.

Human umbilical vein endothelial cells (HUVECs) were a gift from Dr. Shahin Rafii (Weill Cornell Medicine) and were grown in Iscove’s Modified Dulbecco’s Medium (Thermo Fisher Scientific) with 20% Gibco KnockOut Serum Replacement (Thermo Fisher Scientific) until they were used in the cell-survival experiments.

### Isolation of EVs

Conditioned media was created by 48-h incubation of adherent BCi-NS1.1 cells in 25 mL of BEGM lacking BPE in Falcon T-175 cm^2^ flasks (Corning Life Sciences). This media was harvested and centrifuged at 2000X g for 30 min at 4 °C. The supernatant media was then transferred to Centricon Plus-70 Centrifugal Filter Devices with 100 K nominal molecular weight limit (Millipore, Burlington, MA, USA), where it was concentrated down to approximately 10 mL, in accordance with the manufacturer’s instructions; these filter devices use a regenerated cellulose membrane. The concentrated media was then transferred to a sterile, thin-walled polyallomer tube (Beckman Coulter, Brea, CA, USA), and 0.5 volumes of Invitrogen Total Exosome Isolation from Cell Culture Media Reagent (Invitrogen, Carlsbad, CA, USA) was added; isolation of EVs was then performed in accordance with the manufacturer’s protocol—specifically, the conditioned media was mixed with the reagent and incubated overnight at 4 °C, followed by centrifugation at 10,000X g for 1 h. The resultant EV pellet was resuspended in PBS and further characterized or used in experiments. This same process was used to isolate EVs from H1975 and N417 cell-conditioned media.

### Characterization of EVs

The number, size, and concentration of the isolated EV particles were analyzed using the NanoSight NS500 nanoparticle characterization system with Nanoparticle Tracking Analysis software, in accordance with the manufacturer’s instructions (version 2.3, NanoSight, Malvern Instruments, Malvern, Worcestershire, UK).

The protein concentrations of the isolated EV samples and of BCi-NS1.1 cell extracts were determined using the Pierce BCA Protein Assay Kit (Thermo Fisher Scientific), in accordance with the manufacturer’s instructions.

For immunoblotting, EV suspensions and cell extracts were run on 4–12% Bis–Tris NuPAGE mini gels (Thermo Fisher Scientific) and then transferred to polyvinylidene fluoride membranes. The membranes were then incubated for 1 h at room temperature in 5% blocking reagent made with nonfat milk in PBS containing 0.1% Tween-20 (Sigma-Aldrich) (PBS-T). Membranes were probed overnight with primary antibodies dissolved in blocking reagent. The next day, membranes were washed with PBS-T 3 times for 5 min each and then incubated for 1 h at room temperature with anti-rabbit or anti-mouse secondary antibodies conjugated to horseradish peroxidase dissolved in blocking reagent. Membranes were then washed again with PBS-T 3 times for 5 min each. Antibodies were visualized after the addition of SuperSignal chemiluminescent horseradish peroxidase substrate detection reagents (Thermo Fisher Scientific) by exposure to x-ray film.

The following primary antibodies were used for immunoblotting of EV preparations and cells: mouse monoclonal anti-human CD63 (NK1/C3) (sc-59286; 1/1000; Santa Cruz Biotechnology, Dallas, TX, USA), mouse monoclonal anti-human HSP90 (SMC-107A/B; 1/833.3; StressMarq Biosciences, Victoria, British Columbia, Canada), mouse monoclonal anti-human Hsc70 (B-6) (sc-7298; 1/200; Santa Cruz Biotechnology), mouse monoclonal anti-human TSG101 (#612,697; 1/200; BD Biosciences, San Jose, CA, USA), rabbit monoclonal anti-human/monkey Calnexin (C5C9) (#2679; 1/1000; Cell Signaling Technology, Danvers, MA, USA), mouse monoclonal anti-human Alix (1A12) (sc-53540; 1/200; Santa Cruz Biotechnology), and rabbit anti-human β-actin (ab822750; 1/5000; Abcam, Cambridge, MA, USA).

To analyze EV preparations by electron microscopy, 5 µL of sample dissolved in PBS was applied to copper mesh formvar grid discs. After 20 min at room temperature, liquid was removed from the discs by absorption onto cellulose filter paper. After this, 50 µl of 1% glutaraldehyde was added to each disc and samples were fixed in this way for 5 min at room temperature. The discs were then placed onto 100 µl drops of distilled water for 5 min at room temperature, and then removed from the water drop; this process was repeated one more time. The samples were then negatively stained by adding 50 µl of 2% uranyl acetate to each disc and incubating for 10 min at room temperature. The uranyl acetate was then removed from the discs by absorption onto cellulose filter paper. The discs were then air-dried for 10 min at room temperature. The samples were then observed using a Jeol 1200 EX Transmission Electron Microscope (Jeol, Peabody, MA, USA) housed at Memorial Sloan Kettering Cancer Center.

We have submitted all relevant data of our experiments to the EV-TRACK knowledgebase (EV-TRACK ID: EV200169) (Van Deun J, et al. *EV-TRACK: transparent reporting and centralizing knowledge in extracellular vesicle research.* Nature methods. 2017;14(3):228–32).

### Exposure of BCi-NS1.1 Cells to CSE

CSE was prepared, standardized, stored, and used as previously described^10^. Briefly, CSE was produced by drawing all of the smoke resulting from the burning of one Marlboro Red commercial cigarette through 12.5 ml of BEBM medium. After filtration (0.2 μm pore size), absorbance was measured with a spectrophotometer. To normalize the CSE concentration across experiments, the absorbance was measured at 320 nm and an optical density of 1 was defined as 100%. CSE was diluted with BEBM medium to a working concentration of 32% and then frozen in single use aliquots at − 20 °C. The 32% CSE dissolved in BEBM was added to BEGM lacking BPE to achieve a final concentration of 6% CSE. BCi-NS1.1 cells were then incubated in this media for 48 h before EVs were isolated as above and then resuspended in equal volumes of PBS.

The number of nanoparticles in each sample was quantified using the NanoSight NS500 nanoparticle characterization system with Nanoparticle Tracking Analysis software. Three characterization videos were obtained for each sample, and the average of the nanoparticle concentrations was calculated. All analysis settings were kept constant for each sample and between samples in a given experiment. We then calculated the ratio of nanoparticles to the number of Trypan Blue-negative (i.e. live) BCi-NS1.1 cells (counted as described above) in the flasks at the time the conditioned media was collected.

### HUVEC survival assay

An equal number of HUVECs per well were plated into 384-well microtiter plates (Corning Life Sciences), with roughly 400–500 cells per experiment. EVs were added to each well at a final concentrations of 0.1 μg/mL, 1 μg/mL, or 10 μg/mL, and the cells were then incubated at 37 °C for 48 h in a total volume of 40 μL of Iscove’s Modified Dulbecco’s Medium with 20% serum replacement. For certain experiments, various concentrations of the VEGFR1-blocking antibody IMC-18F1 (ImClone, New York, NY), the VEGFR2-blocking antibody IMC-C11 (ImClone), the FGFR1 small-molecule inhibitor SSR128129E (#S7167; Selleck Chemicals, Houston, TX, USA), or the Akt small-molecule inhibitor MK-2206 (#S1078; Selleck Chemicals) were also added to the wells together with the EVs. After 48 h, 40 µL of 2 × CyQUANT detection reagent (Thermo Fisher Scientific) was added to each well, in accordance with the manufacturer’s instructions, and the HUVECs were again incubated at 37 °C for 2 h. The resulting number of green fluorescent cells per well was then manually counted using images taken by a Nikon Ti-U inverted fluorescence microscope with digital site camera (Nikon, Tokyo, Japan). All analysis settings of the camera and microscope were kept constant for each sample and between samples in a given experiment.

### VEGF ELISA

BCi-NS1.1 EVs isolated as described above were tested for the presence of VEGFA using an ELISA assay (R&D Biosystems, Minneapolis, MN), in accordance with the manufacturer’s instructions. As a negative control, BEGM without BPE was incubated for 48 h in flasks without cells and then processed by means of the same procedure used to isolate EVs; the product of this was also tested for VEGFA by use of the same ELISA. The ELISA was performed both with and without exposure of the EVs and negative control to RIPA buffer (Sigma-Aldrich) with Complete Protease Inhibitor Cocktail (Roche, Mannheim, Germany), which was used to lyse the EVs.

### Analysis of VEGFR2 signaling protein activation in HUVECs

In total, 1.5 × 10^5^ HUVECs were seeded into a 12-well plate using an endothelial cell growth medium (Medium 199; Sigma-Aldrich) with 20% (v/v) FBS, 20 μg/mL endothelial cell growth supplement (Hallway), 1% (v/v) antibiotics (Hallway), and 20 units/mL heparin (Sigma-Aldrich). After 24 h, this media was removed and the cells were washed twice with PBS and were then serum-starved for 6 h in media without serum, growth factors, antibiotics, or heparin. Following serum-starvation, the cells were stimulated with either (1) base media (BEBM), (2) “control” EVs, or (3) BCi-NS1.1 EVs (1 μg or 10 μg). After stimulation (1 h or 4 h), the media was aspirated from the cells and the cells were washed once with PBS. Following removal of the PBS, the cells were lysed directly in the dish with 1X NuPAGE LDS Sample Buffer (diluted in RIPA buffer containing Complete Protease Inhibitor Cocktail, Halt phosphatase inhibitor cocktail, and 50 mM Dithiothreitol) and processed for immunoblotting, as described above. Activation of VEGFR2 and downstream signaling pathways (STAT3, Akt, p44/p42 MAPK, and p38 MAPK) were evaluated using both phosphor- and pan-specific antibodies targeted against each protein. The data shown are representative of two independent experiments. The following antibodies were used: rabbit monoclonal anti-human VEGFR2 (55B11) (#2479;1/1000; Cell Signaling Technology), rabbit monoclonal anti-human Phospho-VEGFR2 (Tyr1175) (19A10) (#2478; 1/1000; Cell Signaling Technology), rabbit monoclonal anti-human STAT3 (D3Z2G) (#12,640; 1/1000; Cell Signaling Technology), rabbit monoclonal anti-human Phospho-STAT3 (Tyr705) (D3A7) (#9145; 1/1000; Cell Signaling Technology), rabbit polyclonal anti-human Akt (#9272; 1/1000; Cell Signaling Technology), rabbit monoclonal anti-human Phospho-Akt (Ser473) (D9E) (#4060; 1/1000; Cell Signaling Technology), rabbit polyclonal anti-human p44/42 MAPK (Erk1/2) (#9102; 1/1000; Cell Signaling Technology), rabbit polyclonal anti-human Phospho-p44/42 MAPK (Erk1/2) (Thr202/Tyr204) (#9101; 1/1000; Cell Signaling Technology), rabbit polyclonal anti-human p38 MAPK (#9212; 1/1000; Cell Signaling Technology), and rabbit polyclonal anti-human Phospho-p38 MAPK (Thr180/Tyr182) (#9211; 1/1000; Cell Signaling Technology).

### Statistical analysis

Floating bar graphs were prepared and statistical analyses were performed using GraphPad Prism (GraphPad Software, San Diego, CA, USA). *P* values were calculated using methods suggested by the program for each individual experiment, as reported in the figure legends.

## Results

### Human airway basal cells produce EVs in culture

The BCi-NS1.1 cell line is typically maintained in BEGM, which is composed of BEBM supplemented with several growth factors. We found that the fully supplemented BEGM contained particles that were identified by the Nanoparticle Tracking Analysis software and could therefore interfere with interpretation of our experiments. The vast majority of these particles came from the BPE supplement that was added to BEBM, similar to the manner in which FBS can contaminate other media with microparticles (Supplementary Fig. 1). We therefore grew BCi-NS1.1 cells in fully supplemented BEGM until they reached 70–80% confluence and then replaced the media with BEGM lacking BPE. The cells were then further grown in this media before the media was harvested for isolation of EVs.

**Figure 1:**
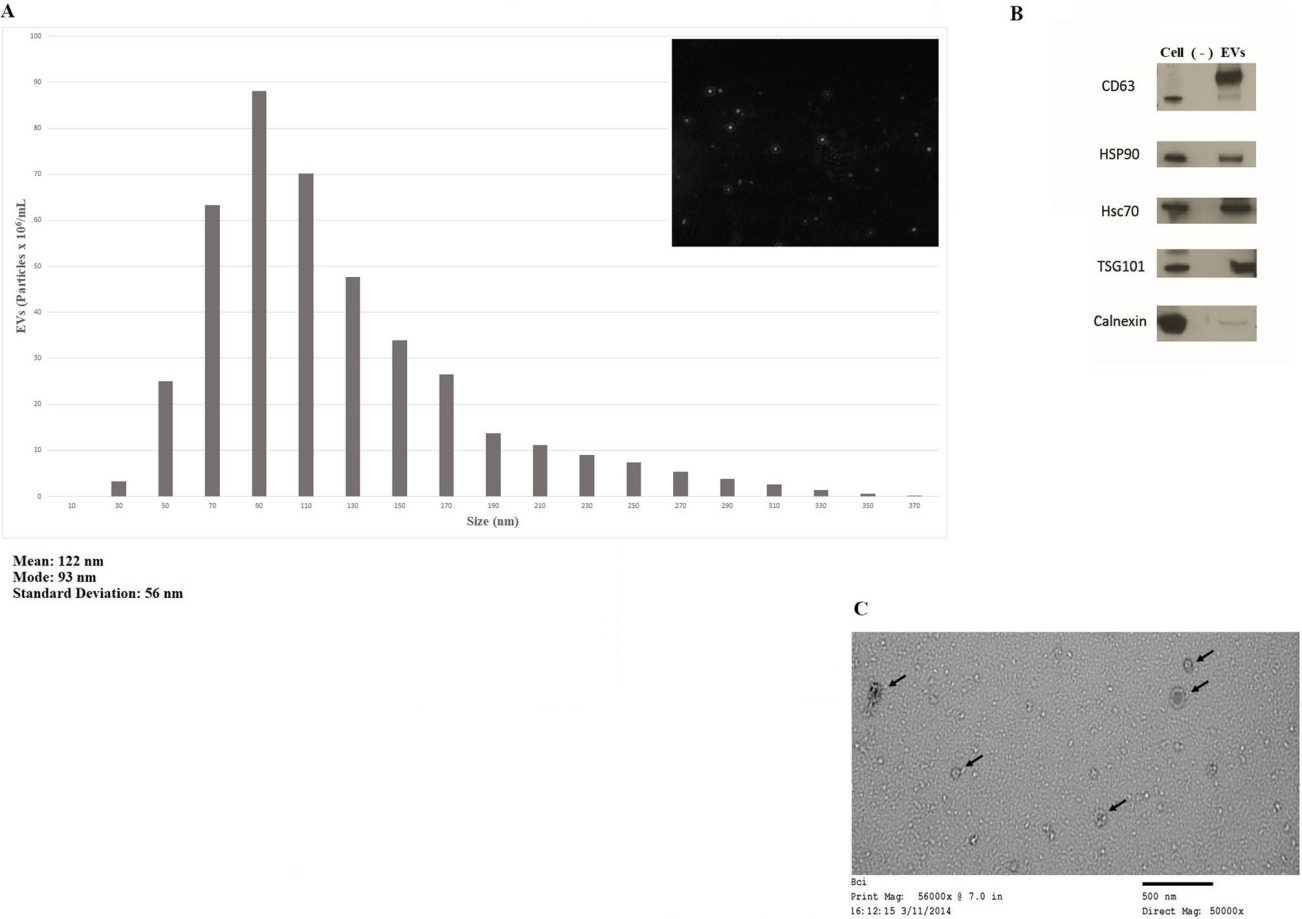
Characterization of EVs produced by immortalized human airway basal cells in culture. (**A**), Representative size distribution histogram of EVs isolated from conditioned media of BCi-NS1.1 cells grown in culture. The NanoSight NS500 machine with Nanoparticle Tracking Analysis software was used to calculate the quantity of particles as well as their size distribution (mean, mode, etc.). A representative image of the particles is shown in the upper right. (**B**), Immunoblots (cropped for clarity and conciseness) of BCi-NS1.1 cell lysates and EVs produced from these cells with antibodies against proteins found in exosomes—CD63, HSP90, Hsc70, and TSG101—as well as Calnexin, which is not typically seen in exosomes. Equal amounts of total protein were loaded into the cell-lysate and EV lanes. As a negative control, BEGM without BPE was incubated for 48 h in flasks without cells and then processed by means of the same procedures used to isolate EVs. A volume equal to that of the EV preparation was loaded onto the lane marked “(—)”. (**C**), Representative image of BCi-NS1.1 EVs (marked by arrows) taken using electron microscopy.

The EVs isolated in this manner were analyzed using the Nanoparticle Tracking Analysis software (Fig. 1A). They had a size distribution roughly consistent with that of exosomes (i.e., < 150 nm). The EVs in these isolates were also enriched for typical exosomal proteins CD63, HSP90, Hcs70, and TSG101. However, they contained little Calnexin protein (Fig. 1B). Examination of isolates using electron microscopy showed characteristic vesicles (Fig. 1C), again supporting the presence of exosomes.

### CSE increases the release of EVs from human airway basal cells

**Figure 2:**
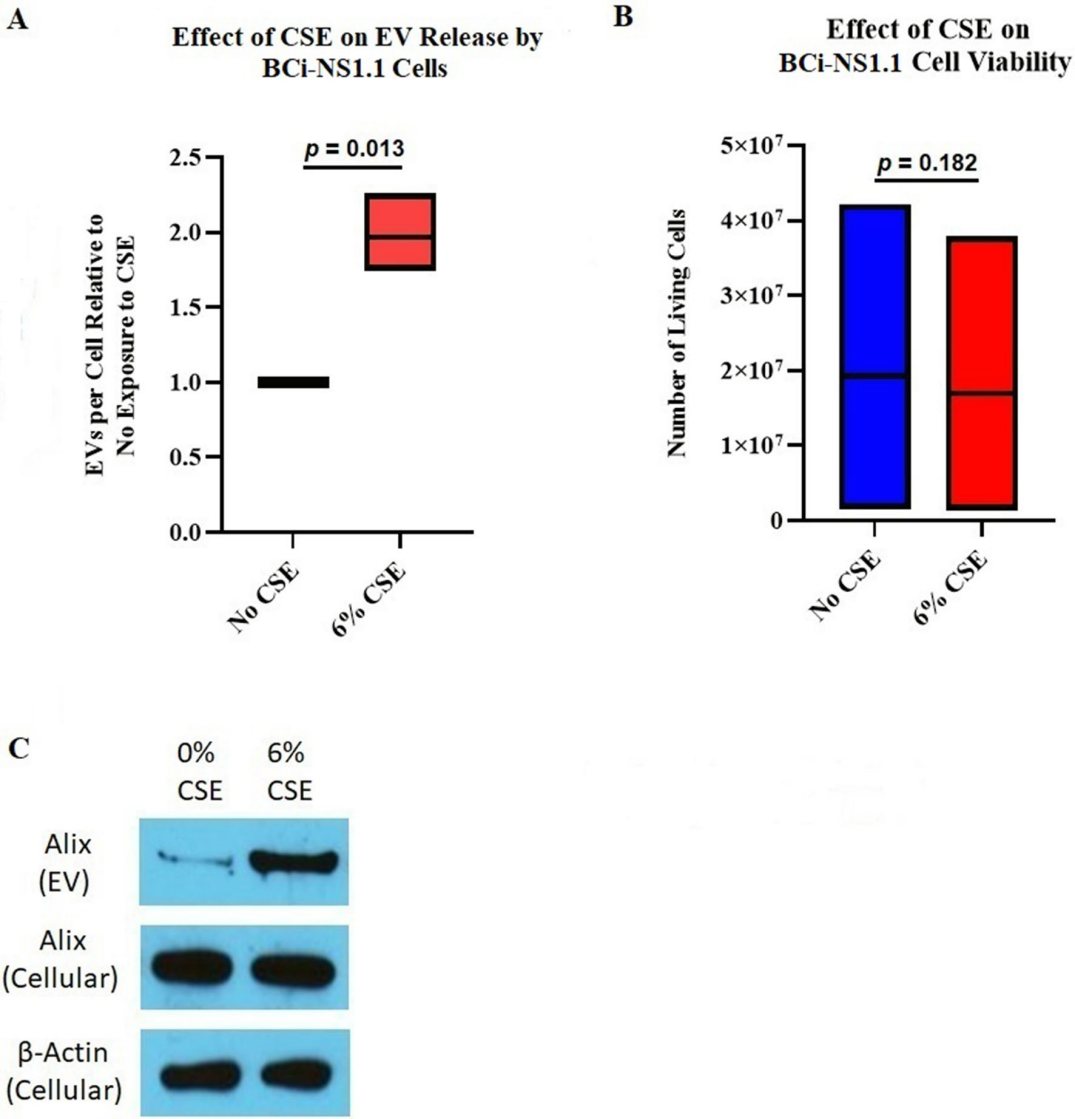
CSE stimulates release of EVs from immortalized human airway basal cells in culture. (**A**)*,* Floating bar graph depicting 3 replicate experiments quantifying EVs isolated from BCi-NS1.1 cells incubated with 6% CSE, compared with those isolated from cells with 0% CSE. The horizontal line inside the floating bar represents the mean of the 3 experiments. Roughly twice as many EVs per cell were seen after CSE exposure; the *P* value (two-tailed) was calculated using the ratio paired *t* test, with *P* ≤ 0.05 considered statistically significant. (**B**)*,* Floating bar graph depicting the number of living BCi-NS1.1 cells just prior to EV isolation in the 3 experiments from A. The horizontal lines inside the floating bars represent the means of the 3 experiments. The *P* value (two-tailed) was calculated using the paired t test, with *P* ≤ 0.05 considered statistically significant. (**C**)*,* Immunoblots (cropped for clarity and conciseness) of the exosomal protein Alix identified in BCi-NS1.1 cell EVs and in cell lysates after exposure to 0% or 6% CSE. Immunoblot of *β*-actin in cell lysates was used as a control. This experiment was repeated 3 times, with representative images shown. Equal amounts of total protein were loaded into each cell-lysate lane, and equal volumes of EV preparations were loaded into each EV lane. In equal volumes of EV preparations, more Alix protein was seen in those from CSE-exposed cells.

We next evaluated whether incubation with CSE would affect the release of EVs from BCi-NS1.1 cells. To do this, we again grew the cells in fully supplemented BEGM until they reached 70%-80% confluence. The cells were then grown for 48 h in the same media but without BPE and with CSE to a final concentration of 6%. Control cells were grown in a similar fashion but without any CSE. The number of adherent cells at the end of the 48-h incubation were counted, and the isolated EVs were quantified using the Nanoparticle Tracking Analysis software. The ratio of EV particles to cells was calculated; this was approximately doubled when the cells were incubated with 6% CSE (Fig. 2A). Of note, the addition of CSE did not result in an equivalent, statistically significant decrease in BCi-NS1.1 cell viability in this assay (Fig. 2B). Additionally, equal volumes of EV isolates analyzed by immunoblotting showed more Alix (a protein enriched in exosomes) in the samples obtained from CSE-exposed cells than unexposed cells, also suggesting that more exosome-enriched EVs were released from cells incubated with CSE. The BCi-NS1.1 cells themselves did not have more Alix protein when they were incubated with 6% CSE (Fig. 2C). Taken together, these data suggest that exposure of human airway basal cells to CSE increases the release of exosome-rich EVs in culture.

### EVs from human airway basal cells promote the survival of human umbilical vein endothelial cells

EVs, and particularly exosomes, are known to interact with target cells and to affect their behavior and cellular processes^11^. For example, cancer cell–derived microvesicles and exosomes can affect proliferation and angiogenesis of endothelial cells^12,13^. We have previously shown that human airway basal cells secrete VEGFA which activates MAPK signaling cascades in endothelial cells via VEGFR2-dependent signaling pathways, in turn resulting in endothelial cell expression of mediators that support proliferation and growth of basal cells^9^. We therefore investigated whether EVs secreted from BCi-NS1.1 airway basal cells affect HUVECs in culture.

We plated HUVECs into wells of a 384-well microtiter plate and incubated them with EVs isolated from cell lines. After 48 h of incubation at 37 °C, we added the CyQUANT detection reagent to each well—this detection reagent contains a green-fluorescent, cell-permeant DNA-binding dye, combined with a background suppression reagent that blocks staining of dead cells or those with compromised cell membranes so that only healthy cells are stained. After cells were incubated for 2 h with this reagent, we counted the number of live cells using fluorescence microscopy.

**Figure 3:**
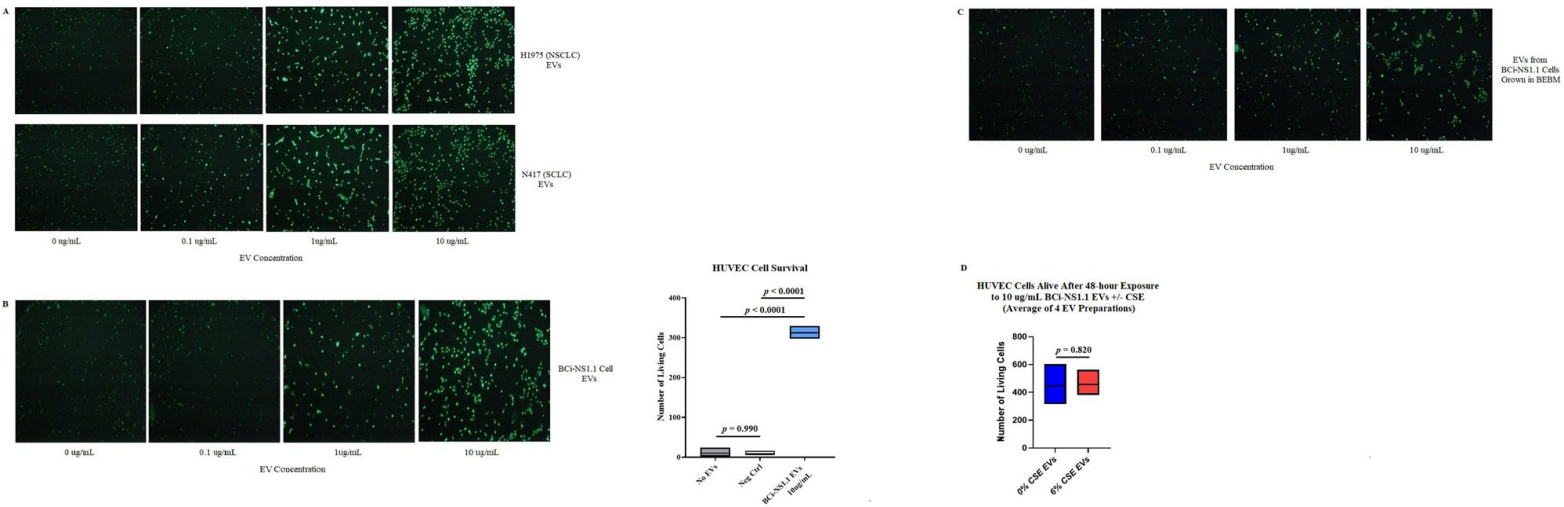
Human airway basal cell EVs promote the survival of HUVECs in culture. (**A**)*,* Images of HUVECs in single wells of a 384-well microtiter plate following 48-h incubation with different concentrations of EVs isolated from the H1975 non-small cell lung cancer and N417 small cell lung cancer cell lines. (**B**), Images of HUVECs in single wells of a 384-well microtiter plate following 48-h incubation with different concentrations of BCi-NS1.1 cell EVs. The live cells were counted under fluorescence microscopy, and the results of 3 replicate experiments using 10 µg/mL EVs is summarized in the floating bar graph to the right. The horizontal lines inside the floating bars represent the means of the 3 experiments. Neg Ctrl, negative control (an equal volume of a preparation from BEGM without BPE incubated for 48 h in flasks without cells and then processed by means of the same procedures used to isolate EVs). Adjusted *P* values correspond to differences between the bars on either end of the horizontal line below them and were calculated using Tukey’s multiple comparisons test, with *P* ≤ 0.05 considered statistically significant. (**C**), The same experiment as in Fig. 4B was performed, except with EVs isolated from BCi-NS1.1 cells grown in BEBM, which is not supplemented with growth factors. (**D**), Floating bar graph of the numbers of living HUVECs after 48-h incubation with 10 µg/mL EVs from BCi-NS1.1 cells exposed to either 0% or 6% CSE. The horizontal lines inside the floating bars represent the means of 4 experiments, each performed with different batches of EV preparations. The *P* value (two-tailed) was calculated using the paired *t* test, with *P* ≤ 0.05 considered statistically significant.

EVs isolated from both non-small cell and small cell lung cancer cell lines promoted the survival of HUVECs in our assay in a concentration-dependent manner (Fig. 3A). This was in keeping with prior studies that have shown proangiogenic effects from EVs released by cancer cells^12,13^. When we incubated the HUVECs with EVs isolated from BCi-NS1.1 cells, we also saw a concentration-dependent increase in survival of endothelial cells. In a negative control in which BEGM without BPE was incubated in tissue-culture flasks without cells and then processed by means of the same procedure used to isolate EVs, survival of HUVECs was not enhanced (Fig. 3B). The enhanced survival of HUVECs was also not attributable to contaminating growth factors in the media the BCi-NS1.1 cells were grown in and from which the EVs were isolated, as EVs isolated from BCi-NS1.1 cells grown in BEBM, which is not supplemented with growth factors, also produced this effect (Fig. 3C).

As our data showed that CSE stimulated the release of more EVs from BCi-NS1.1 cells, we examined whether these CSE-exposed EVs had a differential effect on HUVECs. We found that the CSE-exposed EVs did not further enhance survival of HUVECs in our assay, compared with similar concentrations of EVs not exposed to CSE (Fig. 3D).

### VEGFR2 is involved in human airway basal Cell EV–mediated survival of HUVECs

**Figure 4:**
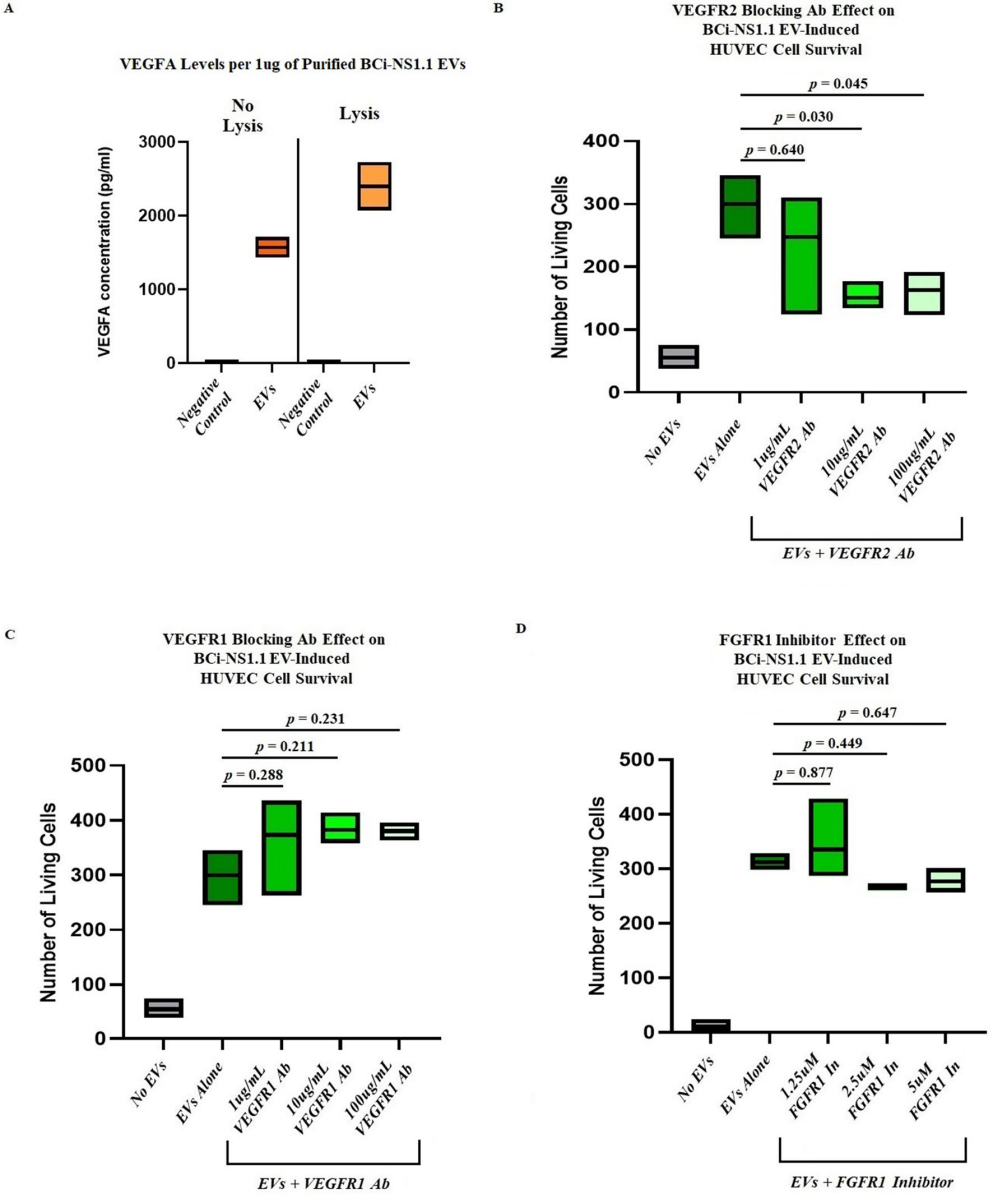
VEGFR2, but not VEGFR1 or FGFR1, is involved in human airway basal cell EV–mediated survival of HUVECs. (**A**), Results of ELISAs, showing the presence of VEGFA in EVs isolated from BCi-NS1.1 cells. Negative control, an equal volume of a preparation from BEGM without BPE incubated for 48 h in flasks without cells and then processed by means of the same procedures used to isolate EVs. The floating bar graphs depict results from 3 batches of isolated EVs, with each batch run in duplicate. The horizontal lines inside the floating bars represent the mean concentrations from the 3 batches. The ELISAs were also performed after EVs were incubated with RIPA buffer to lyse them open. VEGFA was detected both with and without lysis. *B–D*, Floating bar graphs of the numbers of living HUVECs counted after 48-h incubation with either no EVs or 10 µg/mL EVs from BCi-NS1.1 cells in the presence of increasing concentrations of (**B**) VEGFR2-blocking antibody IMC-C11, (**C**) VEGFR1-blocking antibody IMC-18F, and (**D**) FGFR1 small-molecule inhibitor SSR128129E. The horizontal lines inside the floating bars represent the means of 3 experiments; adjusted *P* values correspond to differences between the bars on either end of the horizontal line below them and were calculated using Dunnett’s multiple comparisons test, with *P* ≤ 0.05 considered statistically significant.

We next explored the mechanisms by which BCi-NS1.1 EVs could promote survival of HUVECs. As previous work has shown that these cells produce and secrete VEGFA, we assessed our BCi-NS1.1 EVs for the presence of VEGFA using an ELISA assay. VEGFA was detected in the purified EVs, suggesting that the protein was on the surface of these particles (Fig. 4A). In a negative control of media without BPE incubated in flasks without cells and then processed by means of the same procedure used to isolate EVs, VEGFA was not detected by use of the ELISA. Interestingly, lysis of the EV preparation with RIPA buffer increased the amount of VEGFA detected, suggesting that the protein may also be present inside the vesicles.

As VEGFA is the ligand for both VEGFR1 and VEGFR2, we examined the effect of VEGFR-blocking antibodies on EV-mediated survival of HUVECs. We found that incubation of HUVECs with either a VEGFR1-blocking antibody (IMC-18F1) or a VEGFR2-blocking antibody (IMC-C11) alone, in the absence of EVs, did not promote survival of HUVECs (data not shown). When HUVECs were exposed to BCi-NS1.1 EVs, however, the VEGFR2-blocking antibody, but not the VEGFR1-blocking antibody, significantly decreased the viability of HUVECs in culture (Fig. 4B and 4C, respectively). This suggests that VEGFR2 signaling is important for EV-mediated survival of endothelial cells.

As BCi-NS1.1 cells have also been found to produce fibroblast growth factor (FGF), we examined whether blocking FGF receptor (FGFR) activity would also impair basal cell EV–mediated survival of HUVECs in our assay. After incubation of HUVECs with increasing concentrations of an FGFR1 small-molecule inhibitor (SSR128129E) in the presence of BCi-NS1.1–derived EVs, we did not observe a statistically significant decrease in survival of HUVECs (Fig. 4D).

**Figure 5:**
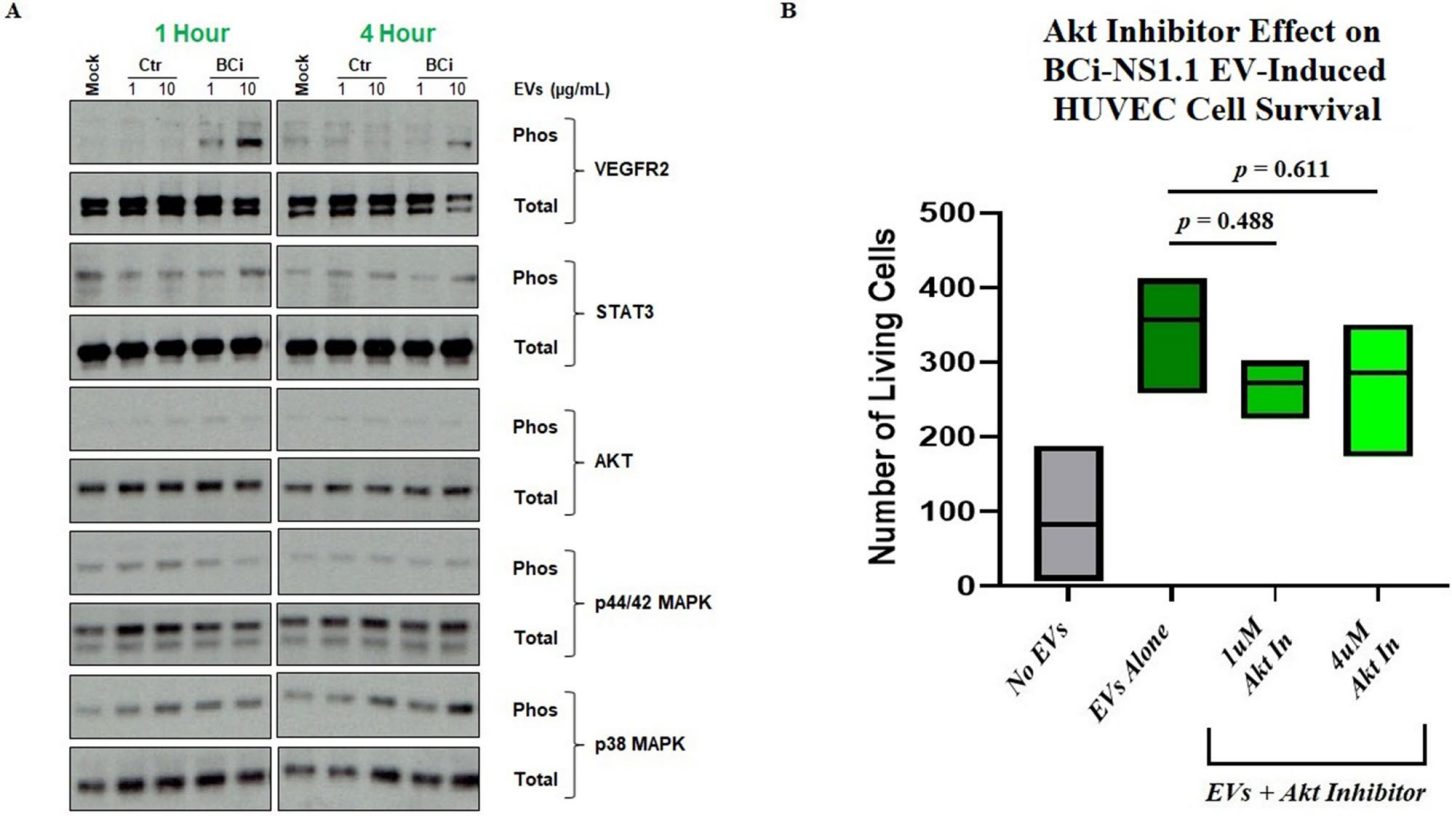
Downstream effectors of human airway basal cell EV–mediated survival of HUVECs. (**A**) Immunoblot of phosphorylated and total VEGFR2, STAT3, Akt, p44/42 MAPK, and p38 MAPK proteins in HUVECs after incubation with BCi-NS1.1 cell EVs or controls for 1 h or 4 h. The experiment was performed in duplicate. BCi, incubation with EVs from BCi-NS1.1 cells; Ctr, incubation with a preparation from BEGM without BPE incubated for 48 h in flasks without cells and then processed by means of the same procedures used to isolate EVs; 1, 1µg of EVs or an equal volume of Ctr; 10, 10µg of EVs or an equal volume of Ctr; Mock, incubation with BEBM media alone. (**B**) Floating bar graph of the numbers of living HUVECs counted after 48-h incubation with either no EVs or 10 µg/mL EVs from BCi-NS1.1 cells in the presence of increasing concentrations of the Akt small-molecule inhibitor MK-2206. The horizontal lines inside the floating bars represent the means of 3 experiments; adjusted *P* values correspond to differences between the bars on either end of the horizontal line below them and were calculated using Dunnett’s multiple comparisons test, with *P* ≤ 0.05 considered statistically significant.

To further confirm that VEGFR2 plays a role in the mechanism of human airway basal cell EV–mediated survival of HUVECs, and to determine what downstream effectors may be involved in this pathway, we examined the levels of phosphorylated VEGF signaling pathway proteins in HUVECs that were exposed to BCi-NS1.1 EVs. HUVECs were serum-starved for 6 h and then incubated with increasing concentrations of BCi-NS1.1 EVs for 1 h or 4 h. The cells were then harvested and lysates were analyzed by immunoblot to assess the relative abundance of phosphorylated candidate proteins (Fig. 5A). Incubation with EVs resulted in an increase in phosphorylated VEGFR2, which was most pronounced at the 1-h time point. However, there was no commensurate increase in the phosphorylation of Akt, STAT3, p44/42 MAPK, or p38 MAPK in our assay, which are known downstream effector proteins in the VEGF pathway. Addition of the Akt inhibitor MK-2206 also did not significantly diminish the effect of BCi-NS1.1 EVs on survival of HUVECs (Fig. 5B).

## Discussion

EVs are secreted from multiple different cell types and have been shown to play an important role in intercellular communication. In this study, we isolated a subset of these vesicles from an immortalized human airway basal cell line that we previously developed to grow in culture. This cell line, BCi-NS1.1, recapitulates many of the same properties of normal basal cells of the human airway epithelium lining the bronchial tree. Thus, we believe that the release of vesicles from these cells is representative of how the cells may function in vivo. The vesicles we isolated appear to be enriched for exosomes, on the basis of their size (as measured using the Nanoparticle Tracking Analysis software), their appearance under electron microscopy, and the abundant presence of exosomal proteins, such as CD63, Alix, and TSG101, as detected by immunoblotting. However, we cannot exclude the possibility that other categories of EVs were also isolated in our preparations or that non-EV-associated proteins were co-precipitated, particularly given our method of isolation (the Invitrogen Total Exosome Isolation from Cell Culture Media Reagent)^14,15^. Nevertheless, our experiments show that human airway basal cells are capable of producing EVs, which likely play an important role in the communication of these progenitor cells with their environment.

Cigarette smoke contains a number of chemicals and carcinogens that are known to affect cells in the respiratory tract, and other cellular stresses have also been shown to effect vesicle release. For example, hypoxia can result in a similar doubling of the number of EVs secreted by several breast cancer cell lines^16^. An increase in secretion of EVs has been shown to occur from cultured human bronchial epithelial cells exposed to CSE in some studies^17,18^, although not in others^19^. We found that exposure to CSE nearly doubles the number of exosome-enriched EVs released by airway basal cells, as quantified using the Nanoparticle Tracking Analysis software. The vesicles released from airway basal cells in response to cigarette smoke could subsequently interact with the surrounding microenvironment in a paracrine manner, altering it in some way in response to the stress. EVs induced in response to respiratory exposures such as cigarette smoke have previously been reported to do just this, modulating inflammation, thrombosis, tissue remodeling, and endothelial dysfunction and angiogenesis^20^.

Endothelial cells make up one component of the microenvironment that interacts with human airway basal cells, as we have previously described. Specifically, in this prior work, we found that primary human airway basal cells isolated and cultured from bronchial tree brushings of nonsmokers secrete VEGFA into the growth media, which leads to activation of signaling cascades in cultured HUVECs. These activated HUVECs in turn support the growth and proliferation of basal cells. Due to this ability to respond to airway basal cell secreted growth factors, we used HUVECs as a surrogate model for lung-derived endothelial cells in the current study as well^5,9^. The BCi-NS1.1 cells from which EVs were obtained were generated from brushed cells isolated from large airway epithelium. As there is no physical contact between basal epithelial cells and endothelial cells in this region of the airway, we focused on paracrine-mediated mechanisms of communication (which EVs are known for) and stimulated endothelial cells with basal cell-derived EVs to add physiological relevance. In this current study, we identified another aspect of the cross talk between human airway basal cells and endothelial cells: the promotion of survival of HUVECs, specifically via exosome-enriched EVs secreted by basal cells. Interestingly, although exposure to CSE increased the number of vesicles released from BCi-NS1.1 cells, these vesicles themselves were not more potent at promoting survival of HUVECs than were vesicles from cells not exposed to CSE. This suggests that cigarette smoke may enhance survival of endothelial cells by increasing the amount, but not the character, of EVs released. However, as we did not specifically compare the contents of the CSE-exposed and non-CSE-exposed EVs, we cannot be certain that they do not carry different cargo. Cigarette smoke itself has been shown to affect lung endothelial cells in a number of ways, such as contributing to barrier dysfunction, endothelial activation and inflammation, apoptosis, vasoactive mediator production, and vascular remodeling. EVs secreted from airway basal cells in response to cigarette smoke may play a role in ameliorating some of these processes, in part by enhancing survival of endothelial cells, which could be connected to endothelial dysfunction and vascular remodeling, as seen in patients with COPD with pulmonary hypertension^21^.

It was not known from our previous studies whether the VEGFA secreted from airway basal cells was exclusively soluble in media or whether some of this protein was also packaged into EVs. VEGF has been found in EVs secreted from human platelets, glioblastoma cells, and HUVECs themselves^22–24^ In the current study, we found VEGFA in secreted EVs from our airway basal cell line. Of note, when concentrating the EV-conditioned media prior to EV purification, we used Centricon Plus-70 Centrifugal Filter Devices with a 100 kDa membrane nominal molecular weight limit. As the molecular weight of VEGFA is considerably lower than 100 kDa, we believe that the free soluble VEGFA in the conditioned media would have passed into the flow-through during this concentration step, such that the chances of co-purification of free VEGFA with the EVs would be significantly decreased. Interestingly, the amount of VEGFA protein detected was increased when the EVs were lysed with RIPA buffer, suggesting that airway basal cells may secrete EVs with the VEGFA protein on their surface as well as internally.

In the context of human lung disease, prior studies have demonstrated alterations in VEGFA signaling and blood vessel biology in smokers with and without COPD. This includes increased VEGFA expression in the bronchial epithelium of smokers and COPD smokers relative to the normal epithelium^25,26^, increased capillary number in the airway of COPD patients compared to healthy controls^27,28^, and finally increased fragmentation of the basement membrane and altered distribution of vessels in smokers and smokers with COPD compared to healthy non-smokers^29^. Combined, these changes may lead to altered cross-talk between the bronchial epithelium and endothelial cells which may contribute to airway remodeling associated with COPD pathogenesis. Therefore, we believe our study is physiologically relevant and provides information on the impact of cigarette smoke exposure on regulation of basal cell-derived EVs and endothelial function.

Our prior experiments showed that the VEGFA-mediated intercellular signaling between primary cultured basal cells and HUVECs involved activation of VEGFR2 and the MAPK pathway^9^. The exosome-enriched EVs we isolated from our primary airway basal cell line also exerted their effect on the survival of HUVECs via VEGFR2 specifically, as incubation with these EVs led to increased phosphorylation of VEGFR2 in HUVECs, whereas blocking this receptor diminished the ability of the EVs to enhance survival of HUVECs. Notably, neither blocking VEGFR1 nor inhibiting FGFR on HUVECs significantly affected the enhancement of their survival in response to airway basal cell EVs.

Interestingly, although VEGFR2 is involved in airway basal cell EV–mediated survival of HUVECs, the downstream effectors of this phenomenon are unclear, as we observed no increase in Akt, STAT3, p44/42 MAPK, or p38 MAPK phosphorylation in EV-treated HUVECs. These results were surprising given the role of these proteins, particularly Akt, in the survival of endothelial cells^30,31^, and also given that prior experiments showed that cultured primary human airway basal cell–conditioned media triggered at least p44/42 MAPK and p38 MAPK phosphorylation in HUVECs^9^. Our results would suggest that EVs from airway basal cells can activate VEGFR2 signaling in ways that are specific and distinct from those of soluble secreted VEGFA. Consistent with the results of our phosphorylation experiment, the use of an Akt inhibitor (MK-2206) did not significantly abrogate EV-mediated survival of HUVECs. These results may be due to limitations in our assays, however, such as the presence of additional phosphorylation sites within the effector proteins tested that were not recognized by the antibodies used in the experiment, our choice of Akt inhibitor or the concentrations of it that were used, or the specific time points we looked at in our phosphorylation assay. Indeed, kinetics are crucial when studying signaling activation and maximal activation of Akt and ERK phosphorylation in HUVECs may occur just 5–15 min after exposure to VEGFA.^32^ However, it is also possible that an alternative VEGFR2-dependent downstream response is involved in promoting survival of endothelial cells in response to airway basal cell EVs. For example, we did not examine the calcineurin-NFAT signaling pathway, which is also activated by VEGFR2^33,34^ and promotes angiogenesis of endothelial cells^35,36^. Interestingly, this axis in the pulmonary endothelium has also been found to be important for the development of lung metastases in the premetastatic niche of the lung^37^. Future experiments could focus on the effect of airway basal cell EVs on this pathway in lung endothelial cells.

In conclusion, we have isolated exosome-enriched EVs from an immortalized human airway basal cell line that recapitulates the multipotent differentiation capacity of primary airway epithelial basal cells. The secretion of these vesicles is enhanced by exposure to CSE, the contents of which these basal cells likely come into contact with in active smokers. The released vesicles are capable of signaling to endothelial cells in the microenvironment and enhance the survival of endothelial cells in our assay. This phenomenon involves VEGFR2 activation, likely at least in part via VEGFA carried in the EVs. The exact mechanism of downstream signaling from VEGFR2 has not been clearly elucidated. Future experiments will be needed to address this, as well as to examine what other effects airway basal cell EVs have on endothelial cells and other components of their microenvironment, in both normal and diseased states such as COPD and malignancy.

## Supplementary Information

Supplementary Information
