# Supplementary material for: Extracellular vesicles from human airway basal cells respond to cigarette smoke extract and affect vascular endothelial cells
Source: Sci Rep. 2021 Mar 17;11:6104. doi: 10.1038/s41598-021-85534-6 (PMC7969738; doi:10.1038/s41598-021-85534-6)

Extracellular Vesicles from Human Airway Basal Cells Respond to Cigarette Smoke Extract and Affect Vascular Endothelial Cells

Ashish Saxena, MD, PhD[1*], Matthew S. Walters, PhD[2,3], Jae-Hung Shieh, PhD[1], Ling-Bo Shen, MS[1], Kazunori Gomi, PhD[2], Robert J. Downey, MD[4], Ronald G. Crystal, MD[2], Malcolm A.S. Moore, DPhil[1]

[1] Department of Cell Biology, Sloan Kettering Institute, Memorial Sloan Kettering Cancer Center, New York, New York

[2]Department of Genetic Medicine, Weill Cornell Medicine, New York, New York

[3]Department of Medicine, Division of Pulmonary, Critical Care, & Sleep Medicine, University of Oklahoma Health Sciences Center, Oklahoma City, Oklahoma

[4]Thoracic Service, Department of Surgery, Memorial Sloan Kettering Cancer Center, New York, New York

Supplementary Figure 1

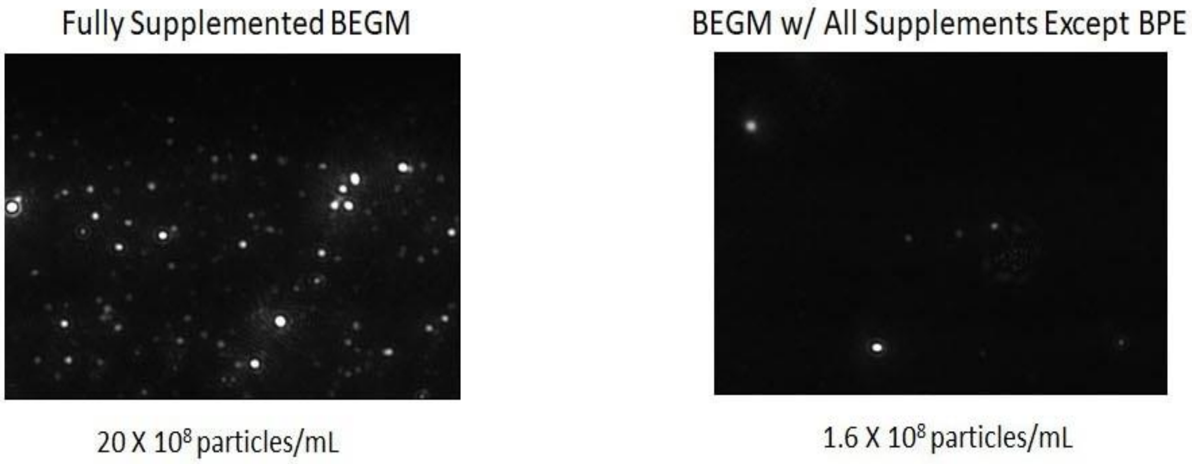

Supplementary Figure 1. The BPE supplement in BEGM contains contaminating microparticles. Representative images of microparticles present in fully supplemented BEGM and those seen in BEGM without BPE are shown. BPE appears to contain the vast majority of microparticles. Images were obtained using the NanoSight NS500 machine with Nanoparticle Tracking Analysis software.

Figure 1B
Full Immunoblots

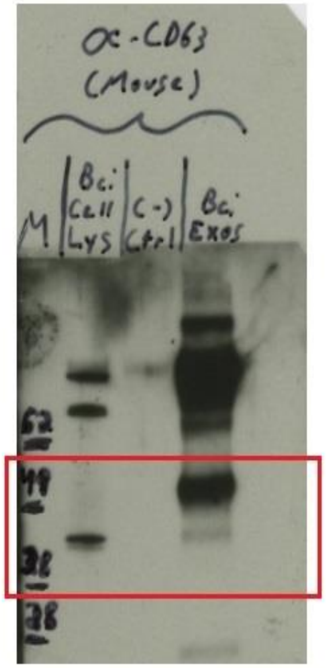

Anti-CD63 Blot

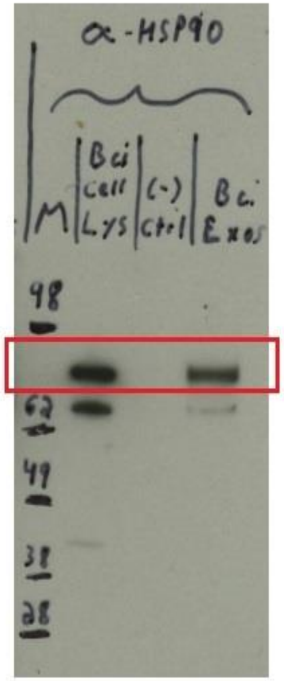

Anti-HSP90 Blot

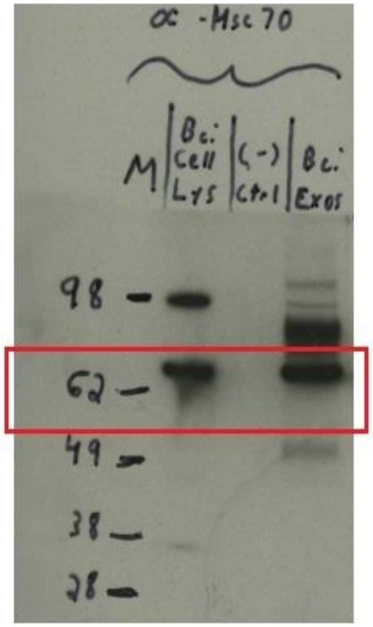

Anti-Hsc70 Blot

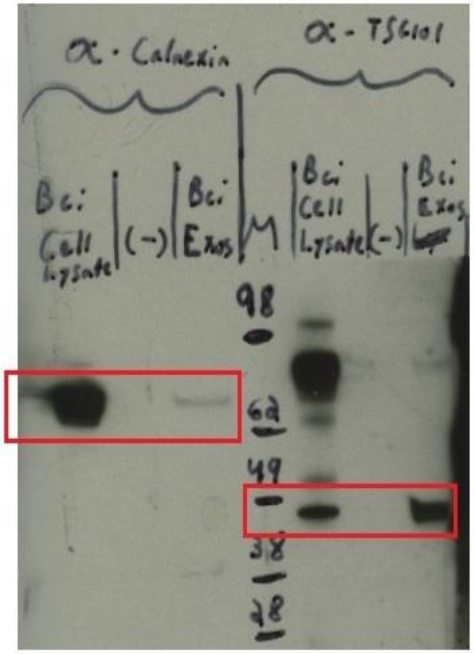

Anti-Calnexin and Anti-TSG101 Blots

Red rectangles denote bands that were cropped for figure

Numbers under "M" column denote molecular weight in kilodaltons

Figure 2C
Full Immunoblots

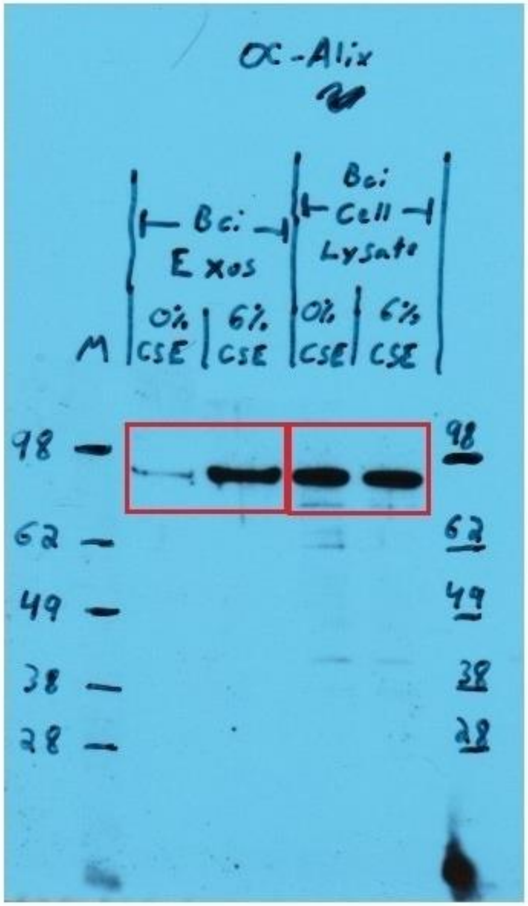

Anti-Alix Immunoblot

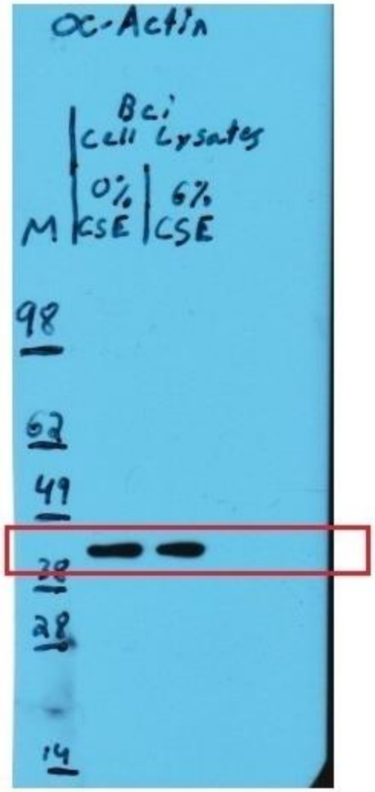

Anti-Actin Immunoblot

Red rectangles denote bands that were cropped for figure

Numbers under "M" column denote molecular weight in kilodaltons

Figure 5A
Full Immunoblots

Red rectangles denote bands that were cropped for figure

A = Phosphorylated VEGR2 Immunoblot
C = Phosphorylated STAT3 Immunoblot
3/4 = 1 hour and 4 hour incubation with Bci-NS1.1 cell EVs

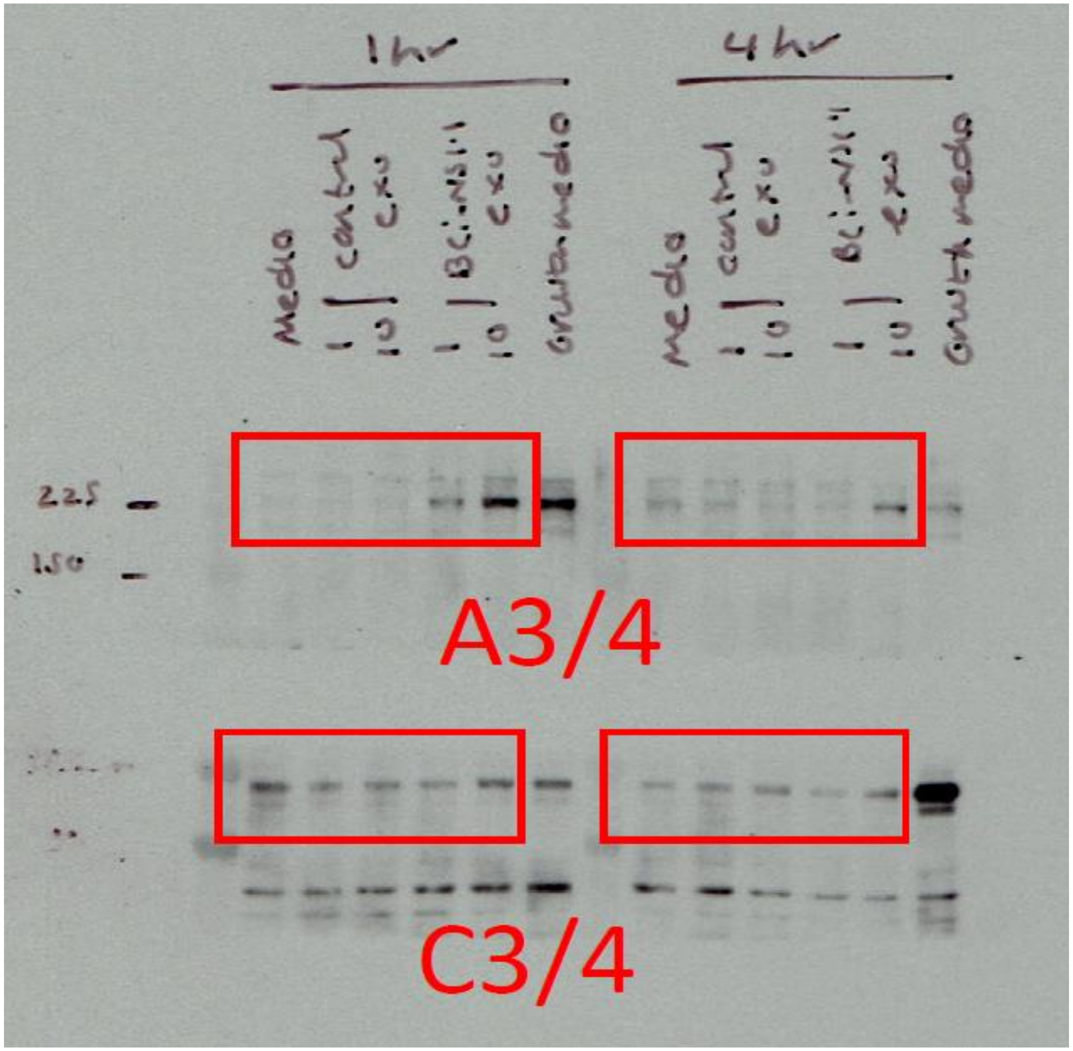

B = Total VEGR2 Immunoblot

D = Total STAT3 Immunoblot

3/4 = 1 hour and 4 hour incubation with Bci-NS1.1 cell EVs

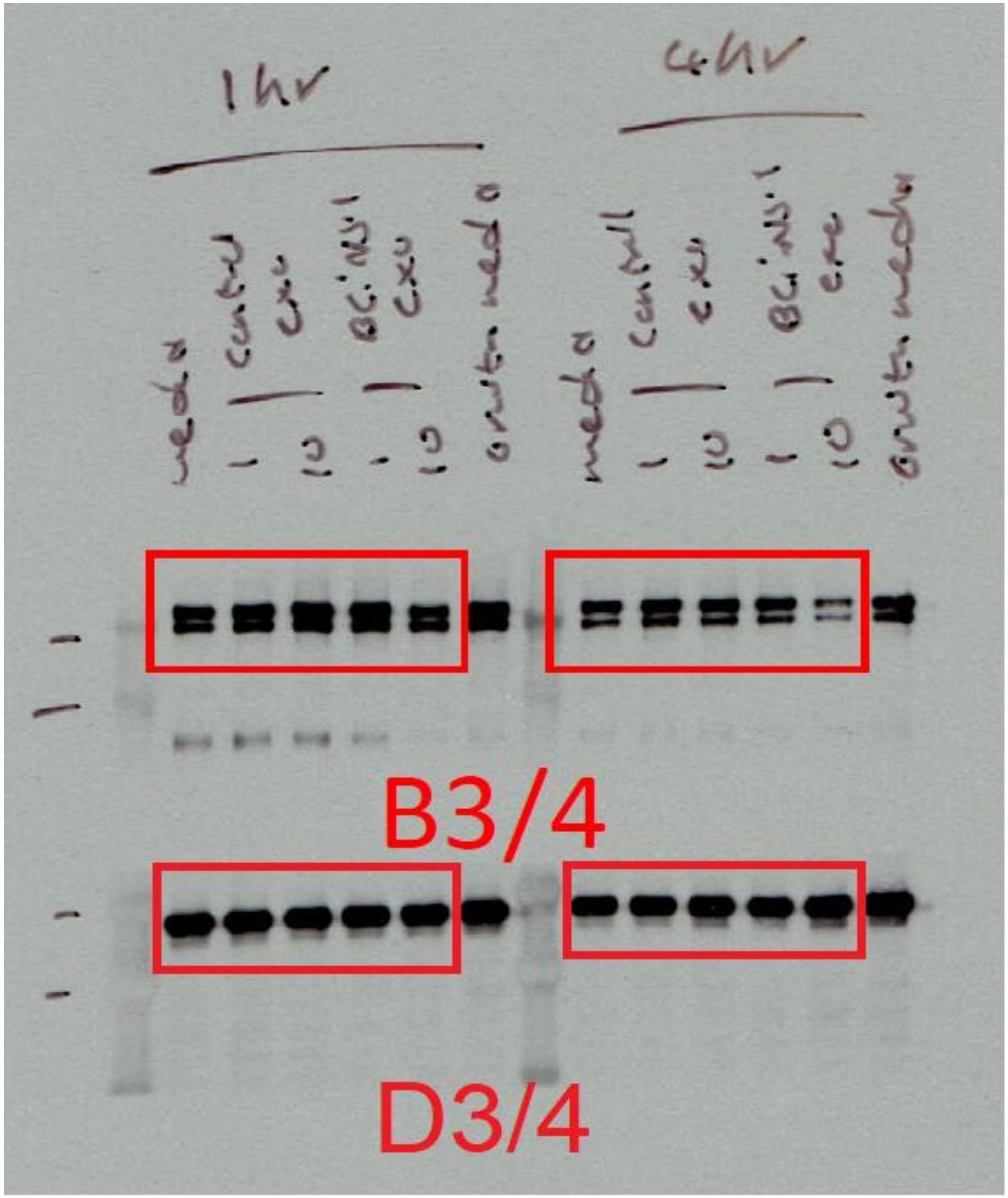

E = Phosphorylated Akt Immunoblot

3/4 = 1 hour and 4 hour incubation with Bci-NS1.1 cell EVs

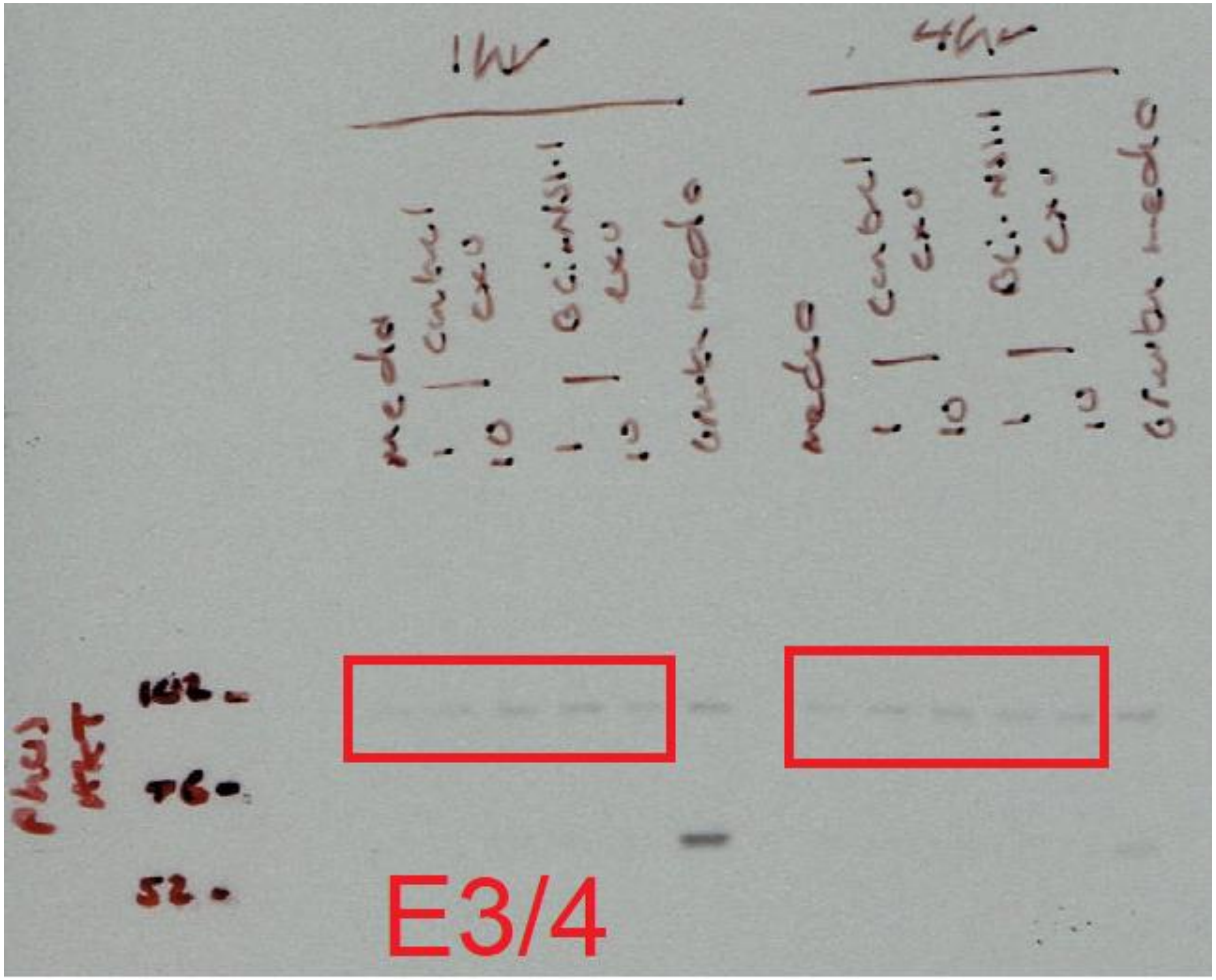

F = Total Akt Immunoblot
H = Total p44/42 MAPK Immunoblot
3/4 = 1 hour and 4 hour incubation with Bci-NS1.1 cell EVs

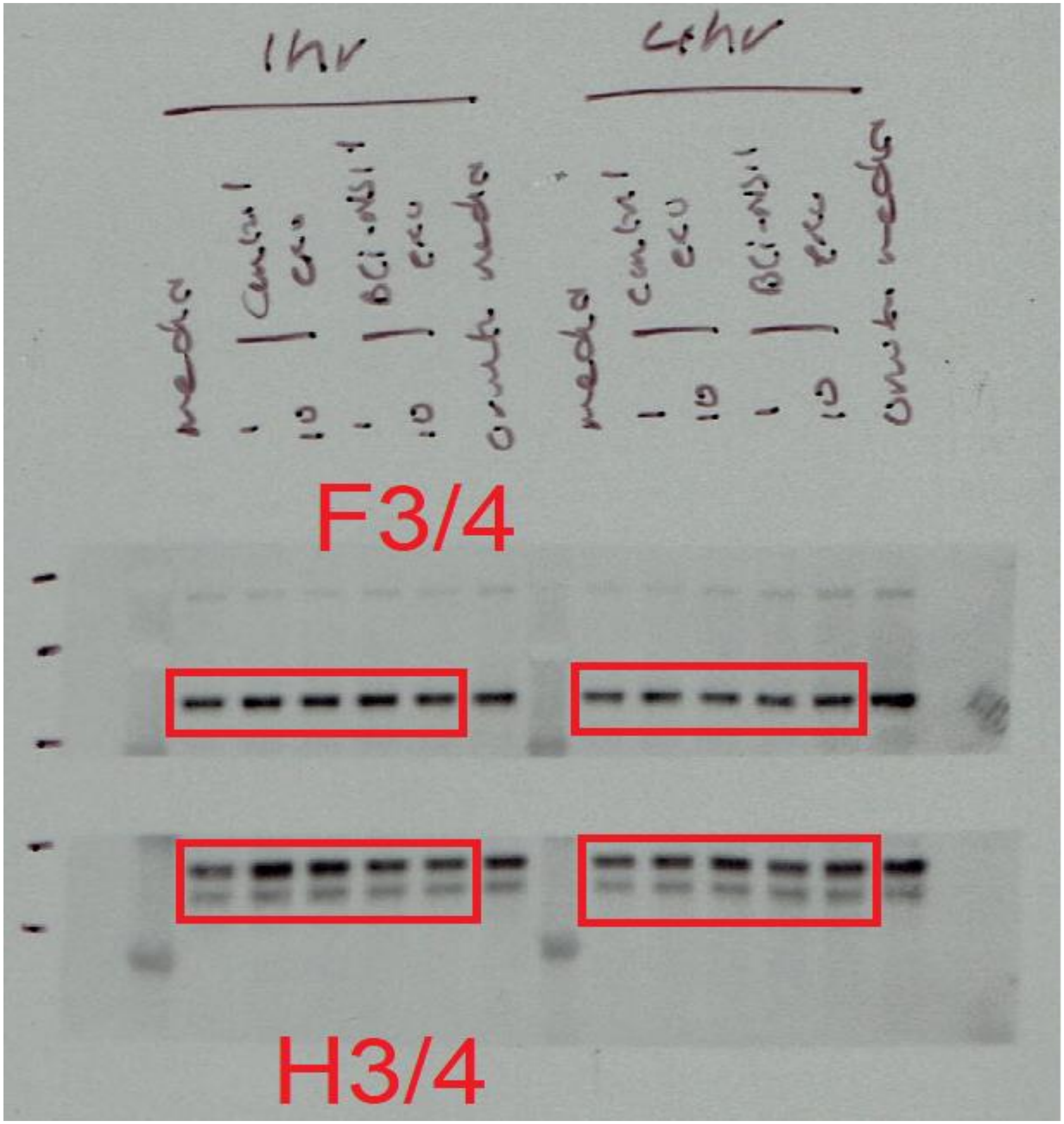

G = Phosphorylated p44/42 MAPK Immunoblot
3/4 = 1 hour and 4 hour incubation with Bci-NS1.1 cell EVs

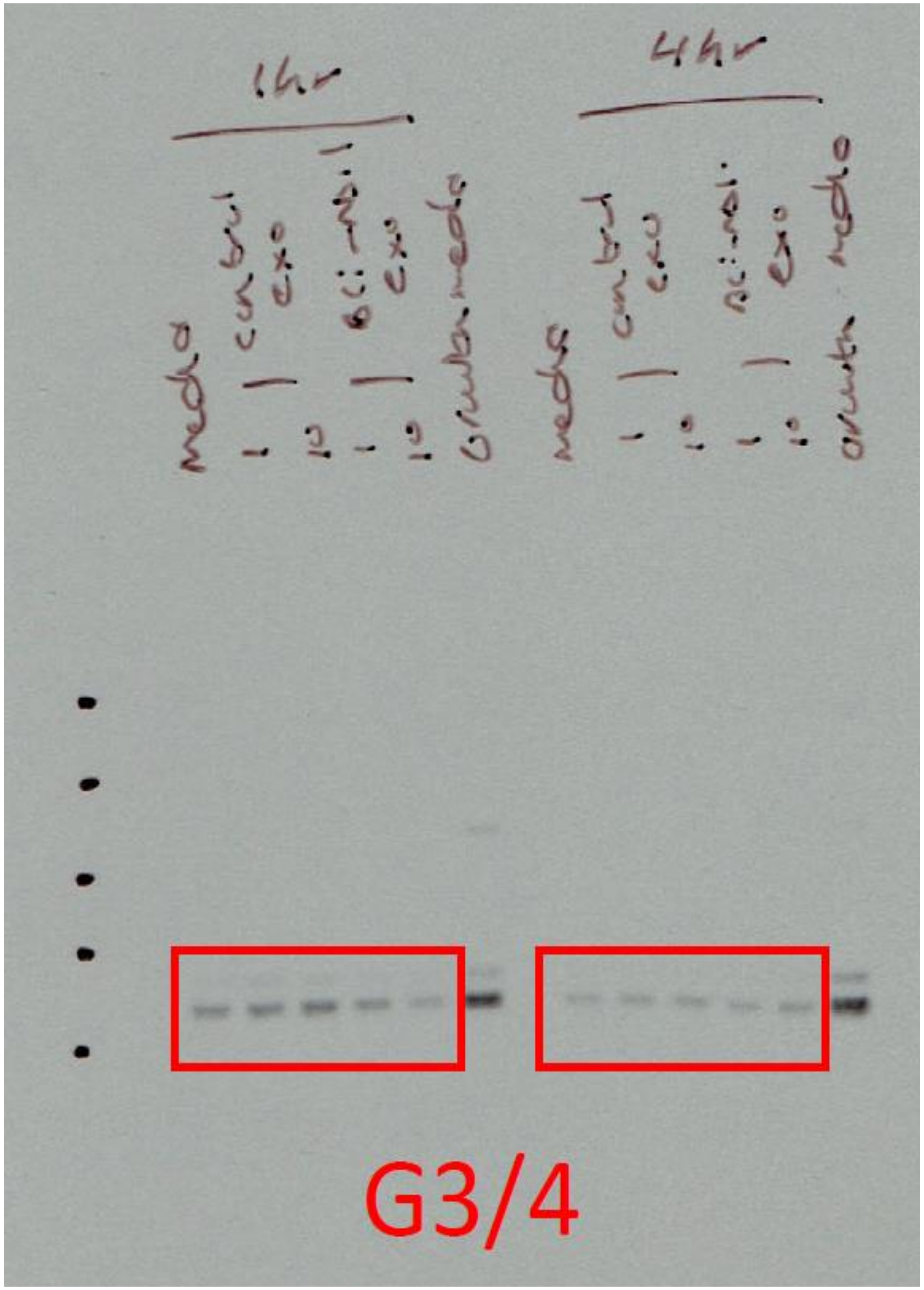

I = Phosphorylated p38 MAPK Immunoblot

3/4 = 1 hour and 4 hour incubation with Bci-NS1.1 cell EVs

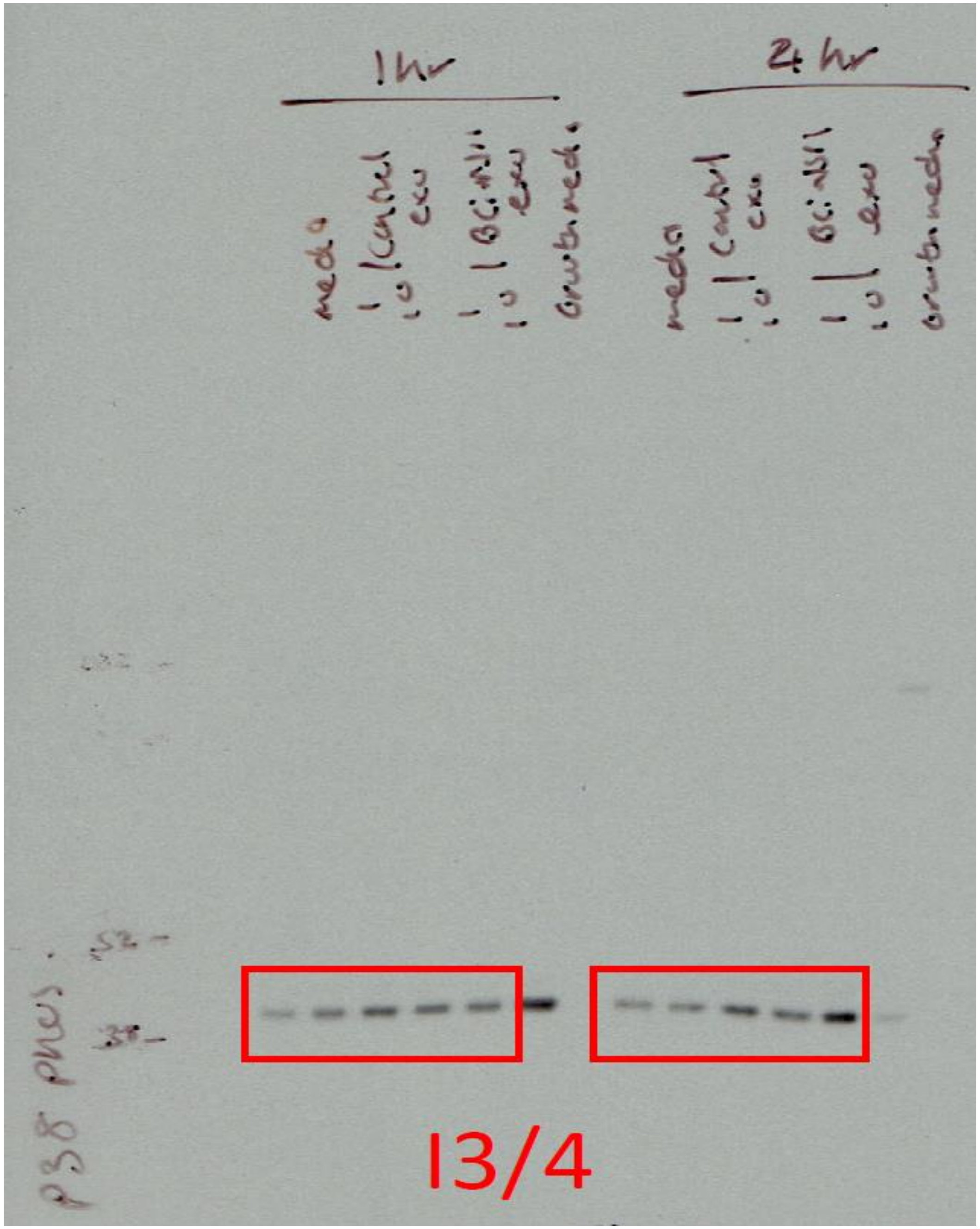

J = Total p38 MAPK Immunoblot

3/4 = 1 hour and 4 hour incubation with Bci-NS1.1 cell EVs

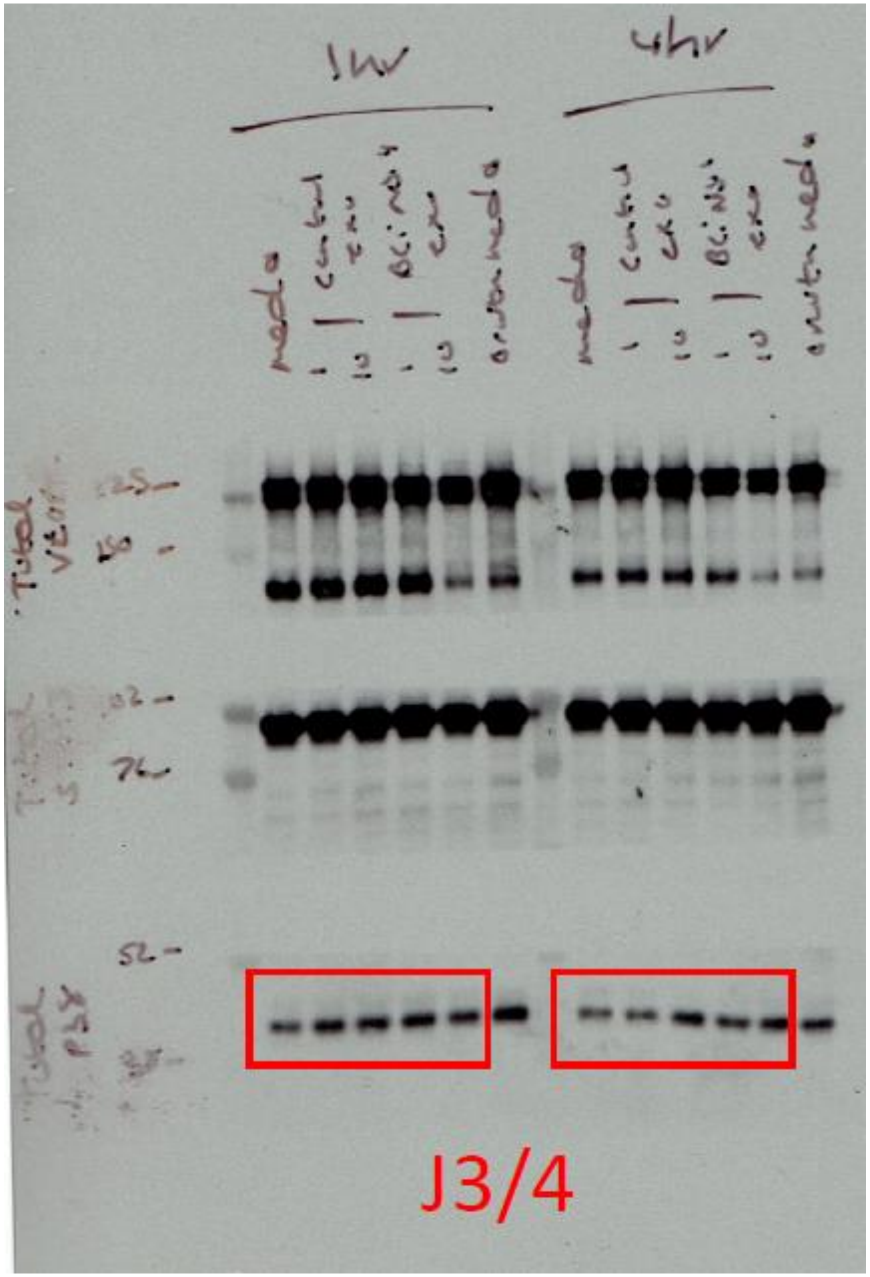

Supplement: Supplementary file 1 — Supplementary Information [file 41598_2021_85534_MOESM1_ESM.pdf]
